# Association Between Dietary Fiber Intake and All-Cause and Cardiovascular Mortality in Middle Aged and Elderly Adults With Chronic Kidney Disease

**DOI:** 10.3389/fnut.2022.863391

**Published:** 2022-04-19

**Authors:** Yu-Jin Kwon, Hye Sun Lee, Go Eun Park, Ji-Won Lee

**Affiliations:** ^1^Department of Family Medicine, Yongin Severance Hospital, Yonsei University College of Medicine, Yongin, South Korea; ^2^Biostatistics Collaboration Unit, Department of Research Affairs, Yonsei University College of Medicine, Seoul, South Korea; ^3^Department of Family Medicine, Gangnam Severance Hospital, Yonsei University College of Medicine, Seoul, South Korea

**Keywords:** dietary fiber, chronic kidney diseases, mortality, Korean Genome and Epidemiology Study, cardiovascular mortality

## Abstract

**Background and Aims:**

Despite accumulating evidence on the benefits of dietary fiber in the general population, there is a lack of representative data on mortality in patients with chronic kidney disease (CKD). This study examined the role of dietary fiber intake on all-cause and cardiovascular mortality in patients with CKD using representative Korean cohort data.

**Methods:**

The study included 3,892 participants with estimated glomerular filtration rates <60 mL/min/1.73 m^2^ from the Korean Genome and Epidemiology Study. Mortality status was followed by data linkage with national data sources. Nutritional status was assessed using a validated food frequency questionnaire. Dietary fiber was categorized into quintiles (Q). A multivariable Cox proportional hazards regression model was used to calculate hazard ratios (HRs) and 95% confidence intervals (CIs) for all-cause and cardiovascular mortality.

**Results:**

The average daily fiber intake of patients with CKD was 5.1 g/day. During the 10.1-year follow-up period, 602 (149 cardiovascular) deaths were documented. The HR (95% CI) for all-cause mortality in the highest quintile compared with that in the lowest quintile was 0.63 (0.46–0.87) after adjusting for age, sex, BMI, smoking, alcohol intake, exercise, total calorie intake, hypertension, diabetes, and dyslipidemia (*P* = 0.005). The HR (95% CI) for cardiovascular mortality in the highest quintile compared with that in the lowest quintile was 0.56 (0.29–1.08) after adjusting for same confounders (*P* = 0.082).

**Conclusion:**

In conclusion, we observed an inverse association between dietary fiber intake and all-cause mortality in CKD patients. Small increments in fiber intake reduced the risk of all-cause mortality by 37%. This finding highlights the need for inexpensive but important dietary modification strategies for encouraging fiber intake in the Korean CKD population.

## Introduction

Chronic kidney disease (CKD) is a major health concern associated with premature mortality or poor quality of life (1). Globally, 10–15% of the population has CKD (2). Furthermore, the rising incidence of CKD is a leading public health burden in East Asia (3). In Korea, the prevalence of CKD in individuals aged >20 was 8.2% (4). Given the high incidence and prevalence of CKD and an urgent need for disease prevention and management strategies, appropriate dietary intake is essential for patients with CKD (5). Previous studies have suggested that unhealthy dietary patterns may contribute to kidney injury and metabolic derangements that may amplify the risks of cardiovascular disease (CVD), morbidity, and mortality (6, 7). Dietary interventions for CKD have been focused mainly on restricting dietary salt, phosphorus, potassium, and protein intake (8). The role of dietary fiber intake in patients with CKD is less well defined. Furthermore, there is no precise fiber intake recommendation for patients with CKD (9). Fiber is abundant in plant foods, including vegetables, fruits, whole grains, nuts, legumes, and seeds (10). Dietary fiber has various health benefits on gut motility, preventing constipation, lowering blood pressure and cholesterol, regulating blood sugar, reducing body weight, and improving gut microbiota (11–13). Adequate fiber intake could reduce the risk of non-communicable diseases (NCDs), including obesity, diabetes, and CVD, which are associated with CKD progression and mortality (14–16). Recently, several meta-analyses including clinical trials have shown that dietary fiber intake reduces the levels of uremic toxins and inflammatory markers and delays the progression of CKD (1, 17). In observational studies, a higher adherence to healthy dietary patterns rich in fiber, such as the Mediterranean diet is associated with a lower risk of incident CKD (18, 19) and poor adherence to a Dietary Approaches to Stop Hypertension (DASH) diets is associated with higher risk of end stage renal disease (ESRD) in patients with pre-existing CKD (20).

Dietary fiber can be divided into soluble dietary fiber and insoluble dietary fiber, which has different physical and functional properties (21). Soluble fiber has key effect on lowering postprandial glucose response and serum lipids. Whereas insoluble fiber has effects on increasing gut transit time and lowering insulin resistance (22). However, most fiber rich foods contain both soluble and insoluble fiber, which have synergic effects on metabolic health (21).

Patients with CKD have a higher mortality rate than the general population (23). CKD resulted in 1.2 million deaths in 2017 and the global all-age CKD mortality rate increased by 41.5% from 1990 to 2017 (2). Krishnamurthy et al. (8) found that higher dietary fiber intake was associated with decreased mortality in patients with CKD, but not in the non-CKD population. Recently, higher dietary fiber intake appeared to have a protective effect on all-cause mortality in non-diabetic peritoneal dialysis patients (24). However, the findings have been inconsistent among observational studies. Some studies have failed to prove any link between fiber intake and mortality in patients on dialysis (25). A more comprehensive study of the association between total fiber intake and all-cause and cause-specific mortality in patients with pre-existing CKD is needed.

Moreover, racial and ethnic disparities, which may influence disparities in clinical outcomes, are important and unresolved issues in patients with CKD (26). Although several populational studies have shown an association between dietary fiber intake and CKD progression and mortality (27, 28), it is unclear whether this association can be generalized to other ethnic groups, where dietary sources and ranges of fiber intake are considerably different. Therefore, this study aimed to comprehensively evaluate the association between dietary fiber intake and all-cause and CVD mortality in adult Korean patients with CKD using large-scale cohort data.

## Methods

### Study Population

This study used data from population-based cohorts in the Korean Genome and Epidemiology Study (KoGES): KoGES_Ansan and Ansung study (community-based cohort in urban and rural counites), the KoGES_health examinee study (national health examinee registry), and the KoGES_cardiovascular disease association study (community-based cohort in rural counties). Participants were recruited through on-site invitation, mailed letters, telephone calls, media campaigns, or community leader-mediated conferences. In KoGES_Ansan and Ansung study, a total of 10,030 participants aged 40–69 years was voluntarily enrolled at baseline between 2001 and 2002. In the KoGES_health examinee study, a total of 173,357 participants was enrolled at baseline from 2004 to 2013. In the KoGES_cardiovascular disease association study, a total of 28,338 participants was enrolled at baseline from 2005 to 2011. A total of 211,571 participants aged over 40 years was enrolled at the baseline KoGES survey. We excluded participants with missing lifestyle (*n* = 2,231), laboratory test (*n* = 5,853), and nutritional intake (*n* = 14,007) data and implausible daily total calorie intakes (<500 kcal or >6,000 kcal). Excluding 54,530 participants with missing mortality data and 63 participants who died in their year of enrollment, a total of 143,050 participants were selected. Finally, a total of 3,892 participants with an estimated glomerular filtration rate (eGFR) of <60 mL/min/1.73 m^2^ were included in the analysis. Among the 3,892 participants, 602 deaths were documented during the follow-up time. The study population selection process is depicted in Supplementary Figure 1. Written informed consent documents were signed by all participants. The study protocol conformed to the ethical guidelines of the 1975 Declaration of Helsinki. This study was approved by the institutional review boards of Yongin Severance Hospital (IRB Number: 9-2021-0066).

### Covariates

The body mass index (BMI) was calculated as the body weight (in kilograms) divided by the square of the height (in meters). Waist circumference (WC) was measured at the midline level between the inferior margin of the ribs and the superior border of the iliac crest. Anthropometric measurements were performed using validated and standardized protocols. Smoking status was categorized as current smoker, former smoker, and non-smoker. A current smoker was defined as any participant who currently smoked and had smoked at least 100 cigarettes during their lifetime. A former smoker was defined as an adult who had smoked at least 100 cigarettes in their lifetime and had quit smoking. Never smokers were defined as adults who had never smoked or had smoked <100 cigarettes in their lifetime. Alcohol intake status was categorized as current drinker, former drinker, and non-drinker. A regular exerciser was defined as a person who regularly exercised more than once a week. Diabetes was defined as a glycated hemoglobin level of 6.5% or higher, a fasting plasma glucose concentration ≥126 mg/dL after overnight fasting, or the taking of anti-diabetic medications. Dyslipidemia was defined as a total cholesterol level ≥200 mg/dL, triglyceride level ≥150 mg/dL, or the taking of anti-dyslipidemic medications. Hypertension was defined as a systolic blood pressure >140 mmHg, diastolic blood pressure >90 mmHg, or the taking of antihypertensive medications. Blood concentrations of creatinine, blood urea nitrogen, glucose, glycated hemoglobin, total cholesterol, high-density lipoprotein cholesterol (HDL-C), low-density lipoprotein cholesterol (LDL-C), triglyceride, alanine aminotransferase (ALT), and aspartate aminotransferase (AST) were analyzed at the central laboratory. Detailed information on the study protocol is available at the following website: https://www.kdca.go.kr/contents.es?mid=a40504100100.

The eGFR was calculated using the Modification of Diet in Renal Disease (MDRD) Study equation (29) as follows:

GFR (mL/min/1.73 m^2^) = 175 × (S_cr_)^−1.154^ × (Age)^−0.203^ × (0.742 if female) × (1.212 if African American).

CKD was defined as an eGFR of <60 mL/min/1.73 m^2^.

### Mortality Ascertainment

Mortality status was followed by data linkage with national data sources based on the unique personal identification key code system. The KoGES data have been linked to national data sources (Korea National Statistical Office), including death records for evaluating mortality. Participant deaths were tracked to December 2019. The underlying causes of death were based on Korean Standard Classification of Disease (KCD) codes listed in the National Death Index. All-cause mortality included all deaths of specified and unknown causes.

### Dietary Assessment

A food frequency questionnaire (FFQ) consisting of 103 food items was developed for the KoGES survey (30). The FFQ addresses the diet of the past 12 months and has long been the instrument of choice in epidemiologic studies. Carbohydrate, fat, and protein intake were recorded as g/day. Dietary fiber intake (g/day) was classified in quintiles (Q). The protocol and results of a validation study for the FFQ are detailed in previous studies (30, 31).

### Statistical Analysis

Data were analyzed from 2001 through 2013. Participant deaths were tracked to December 2019. The follow-up period for each study participant was calculated as the time from their KoGES initial assessment to either a mortality event or the censoring date. Data are presented as mean ± standard deviation (SD), for continuous variables, or number (%), for categorical variables. Dietary fiber intake was divided into quintiles. Baseline characteristics of the study population according to dietary fiber intake quintiles were compared using one-way analysis of variance, for continuous variables, and the chi-square test, for categorical variables. A Cox proportional hazard spline curve was used to assess the association between dietary fiber intake and all-cause mortality risk. The incidence per 1,000 person-years was calculated for each group. The Kaplan–Meier method with the log-rank test was used to compare cumulative incidence rates for all-cause mortality according to dietary fiber intake quintiles. A multivariable Cox proportional hazards regression model was used to calculate hazard ratios (HRs) and 95% confidence intervals (CIs) for all-cause mortality and CVD mortality in Q2, Q3, Q4, and Q5 with reference to Q1, after adjusting for potential confounding factors. Model 1 was adjusted for age, sex, and BMI. Model 2 was additionally adjusted for smoking, alcohol intake, exercise, and total calorie intake. Model 3 was additionally adjusted for history of hypertension, diabetes, and dyslipidemia. Model 4 was additionally adjusted for baseline eGFR. We performed subgroup analysis to investigate the dietary fiber intake and mortality in CKD patients according to CKD stages, sex and BMI criteria 25 kg/m^2^.

Statistical analyses were conducted using SAS version 9.4 (SAS Institute, Cary, NC, USA) and R package version 4.0.3 (http://www.R-project.org). All statistical tests were two sided, and *P* < 0.05 was considered statistically significant.

## Results

During the median 10.1 (min-max, 0.2–15.9) years of follow-up, there were 602 deaths. Detailed information on the study population according to mortality status is presented in Supplementary Table 1 (according to all-cause mortality). The proportion of men was higher among all-cause mortality cases. The mean age was 68.5 ± 7.3 years for participants with a death event and 61.8 ± 8.0 years for participants without death event, respectively. Participants with death events were more likely to be older, to have a lower BMI, higher WC, higher SBP, lower total cholesterol levels, lower HDL-C levels, higher blood glucose and HbA1c levels, smoke more, and have hypertension and diabetes; however, they were less likely to be non-smokers, exercise regularly, and live in urban areas. Participants with death events consumed less total energy, carbohydrates (g/day), fat (g/day), protein (g/day), minerals, and vitamins. However, the carbohydrate intake proportion was higher than that of those without death events.

Table 1 presents the baseline characteristics of the study population according to quintiles of dietary fiber intake. The mean age of study population was 62.9 ± 8.3 years, and the proportion of men was 38.4% in this study. The mean dietary fiber intake was 5.1 ± 2.6 g/day. Participants with higher dietary fiber intakes were more likely to be men, younger, have a higher BMI, higher WC, higher HDL-C levels, drink more alcohol, exercise more regularly, and live in urban areas; however, they were less likely to smoke. Regarding nutritional status, participants with higher dietary fiber intakes were more likely to have higher total energy intakes and consume carbohydrate (g/day), fat (g/day and %), protein (g/day and %), minerals, and vitamins; however, the carbohydrate proportion (%) of their diet was likely to be lower. *Post-hoc* comparison *P*-values between the five categories are presented in Supplementary Table 2.

**Table 1:** Baseline characteristics of the cohort according to dietary fiber intake (g/day).

	**Q1** **(0.50, 3.01)**	**Q2** **(3.02,4.15)**	**Q3** **(4.16, 5.26)**	**Q4** **(5.27, 6.76)**	**Q5** **(6.77, 27.60)**
*n*	778	779	778	779	778
Sex (male), *n* (%)	241 (31.0)	295 (37.9)	294 (37.8)	333 (42.8)	332 (42.7)
Age, years	65.0 ± 9.0	63.6 ± 8.2	62.3 ± 7.8	62.1 ± 7.9	61.3 ± 7.9
BMI, kg/m^2^	24.5 ± 3.3	24.7 ± 3.0	24.9 ± 3.0	25.0 ± 3.1	25.0 ± 2.9
WC, cm	84.1 ± 9.1	85.1 ± 9.0	85.1 ± 8.8	85.9 ± 8.6	85.5 ± 8.7
SBP, mmHg	127.7 ± 18.5	127.7 ± 17.6	127.3 ± 17.0	127.1 ± 17.6	128.2 ± 16.9
DBP, mmHg	77.0 ± 10.3	77.5 ± 10.1	77.4 ± 10.0	77.7 ± 11.0	78.5 ± 10.4
FBG, mg/dl	105.4 ± 36.1	106.1 ± 40.6	104.7 ± 36.1	102.8 ± 30.4	101.9 ± 33.6
HbA1c, %	6.29 ± 1.17	6.19 ± 1.243	6.17 ± 1.20	6.10 ± 1.14	6.14 ± 1.09
TC, mg/dl	196.2 ± 40.6	197.9 ± 39.8	197.6 ± 40.1	197.3 ± 39.6	198.7 ± 39.1
HDL-C, mg/dl	45.1 ± 12.7	45.9 ± 11.8	46.8 ± 11.8	46.7 ± 12.0	46.5 ± 11.6
LDL-C, mg/dl	119.4 ± 36.5	121.6 ± 36.0	120.6 ± 36.2	119.2 ± 35.5	122.1 ± 35.4
TG, mg/dl	158.6 ± 95.2	152.9 ± 89.0	152.1 ± 94.8	158.3 ± 98.5	152.7 ± 96.7
BUN, mg/dl	20.8 ± 11.0	20.3 ± 8.5	20.2 ± 7.4	20.3 ± 7.8	20.0 ± 7.7
Cr, mg/dl	1.40 ± 1.04	1.34 ± 0.63	1.34 ± 0.72	1.38 ± 0.81	1.33 ± 0.86
eGFR, mL/min/1.73 m^2^	51.0 ± 10.6	52.1 ± 9.6	52.4 ± 9.4	52.2 ± 9.7	53.3 ± 8.6
AST, IU/L	25.3 ± 9.4	25.7 ± 9.4	25.3 ± 9.7	25.6 ± 11.1	25.6 ± 11.4
ALT, IU/L	21.9 ± 12.0	22.6 ± 12.1	22.9 ± 14.3	23.4 ± 13.3	23.8 ± 15.4
**Smoking status**, ***n*** **(%)**
Never smoker	568 (73.0)	559 (71.8)	551 (70.8)	521 (66.9)	540 (69.4)
Former smoker	121 (15.6)	151 (19.4)	173 (22.2)	177 (22.7)	166 (21.3)
Current smoker	89 (11.4)	69 (8.9)	54 (6.9)	81 (10.4)	72 (9.3)
**Alcohol intake**, ***n*** **(%)**
Never drinker	511 (65.7)	465 (59.7)	456 (58.6)	437 (56.1)	452 (58.1)
Former drinker	66 (8.5)	71 (9.1)	57 (7.3)	69 (8.9)	49 (6.3)
Current drinker	201 (25.8)	243 (31.2)	265 (34.1)	273 (35.0)	277 (35.6)
Regular exercise, *n* (%)	253 (32.5)	337 (43.1)	366 (47.0)	381 (48.9)	410 (52.6)
HTN, *n* (%)	211 (27.1)	199 (25.6)	213 (27.4)	213 (27.3)	236 (30.3)
DM, *n* (%)	134 (17.2)	136 (17.5)	136 (17.5)	125 (16.1)	105 (13.5)
Dyslipidemia, *n* (%)	487 (62.6)	504 (64.7)	483 (62.1)	496 (63.7)	485 (62.3)
**Residential area**, ***n*** **(%)**
Urban	359 (46.1)	442 (56.7)	488 (62.7)	535 (68.7)	553 (71.1)
Rural	419 (53.9)	337 (43.3)	290 (37.3)	244 (31.3)	225 (28.9)
Total energy intake, kcal/day	1,177.1 ± 335.6	1,395.9 ± 317.9	1,550.7 ± 363.0	1,732.6 ± 380.0	2,065.4 ± 544.5
CHO (g/day)	226.9 ± 63.6	263.5 ± 60.3	287.2 ± 65.9	314.7 ± 67.7	366.4 ± 87.4
CHO (%)	77.3 ± 6.5	75.7 ± 6.4	74.4 ± 6.0	72.9 ± 6.2	71.6 ± 7.0
Fat, g/day	12.5 ± 9.4	16.7 ± 9.9	20.6 ± 11.1	25.4 ± 12.5	33.2 ± 19.5
Fat, %	9.3 ± 5.3	10.6 ± 5.1	11.7 ± 4.8	12.9 ± 5.0	13.9 ± 5.2
Protein, g/day	32.4 ± 12.0	41.6 ± 11.8	48.7 ± 13.6	57.4 ± 15.4	74.1 ± 25.9
Protein, %	11.0 ± 2.1	12.0 ± 2.2	12.6 ± 2.1	13.3 ± 2.1	14.3 ± 2.7
Na, g/day	0.95 ± 0.46	1.66 ± 0.53	2.16 ± 0.62	2.74 ± 0.46	4.00 ± 1.51
K, g/day	0.96 ± 0.37	1.43 ± 0.36	1.81 ± 0.40	2.27 ± 0.46	3.25 ± 0.98
Ca, mg	191.9 ± 116.9	279.9 ± 130.4	348.2 ± 138.6	438.9 ± 161.8	650.5 ± 303.4
P, mg	492.3 ± 163.1	638.2 ± 156.0	748.9 ± 174.3	883.0 ± 200.6	1,151.9 ± 357.4
Fe, mg	4.4 ± 1.5	6.3 ± 1.5	7.9 ± 1.7	9.7 ± 2.1	14.2 ± 4.9
Vit. A, RE	150.9 ± 88.9	255.5 ± 110.6	343.6 ± 129.5	456.9 ± 158.2	789.0 ± 394.7
Vit. B1, mg	0.51 ± 0.19	0.68 ± 0.20	0.81 ± 0.23	0.98 ± 0.25	1.28 ± 0.40
Vit. B2, mg	0.42 ± 0.21	0.58 ± 0.23	0.71 ± 0.24	0.87 ± 0.28	1.20 ± 0.47
Niacin, mg	7.7 ± 2.9	10.1 ± 2.8	11.9 ± 3.2	14.0 ± 3.7	18.3 ± 6.0
Vit. C, mg	33.6 ± 18.6	58.6 ± 22.0	80.3 ± 27.4	108.6 ± 34.4	170.9 ± 67.0
Zinc, ug	4.6 ± 1.8	5.8 ± 1.8	6.6 ± 1.9	7.9 ± 3.0	10.0 ± 4.1
Vit. B6, mg	0.78 ± 0.23	1.08 ± 0.21	1.30 ± 0.25	1.58 ± 0.29	2.20 ± 0.65
Folate, ug	79.7 ± 26.9	130.3 ± 28.8	171.5 ± 32.9	217.8 ± 43.1	335.6 ± 120.2
Retinol, ug	29.4 ± 32.5	39.2 ± 36.7	49.9 ± 43.8	60.6 ± 47.1	81.4 ± 80.0
Carotene, ug	705.0 ± 417.8	1,263.4 ± 560.6	1,717.2 ± 672.5	2,313.8 ± 839.8	4,132.3 ± 2,182.1
Vit. E, mg	3.6 ± 1.7	5.0 ± 1.7	6.2 ± 2.2	7.8 ± 2.5	11.5 ± 4.7

*BMI, body mass index; WC, waist circumference; SBP, systolic blood pressure; DBP, diastolic blood pressure; FBG, fasting blood glucose; TC, total cholesterol; HDL-C, high density lipoprotein cholesterol; LDL-C, low density lipoprotein cholesterol; TG, triglyceride; BUN, blood urea nitrogen; Cr, creatinine; AST, aspartate transaminase; ALT, alanine transaminase; HTN, hypertension; DM, diabetes mellitus; Ca, calcium; P, phosphorus; Fe, ferrous; Vit, vitamin; RE, retinol equivalents*.

*The proportions of carbohydrate, protein, and fat intakes were calculated as carbohydrate intake (g/day), protein intake (g/day) ×4 kcal/total energy intake (kcal/day) ×100, and fat intake (g/day) ×9 kcal/total energy intake (kcal/day) ×100, respectively*.

**Figure 1:**
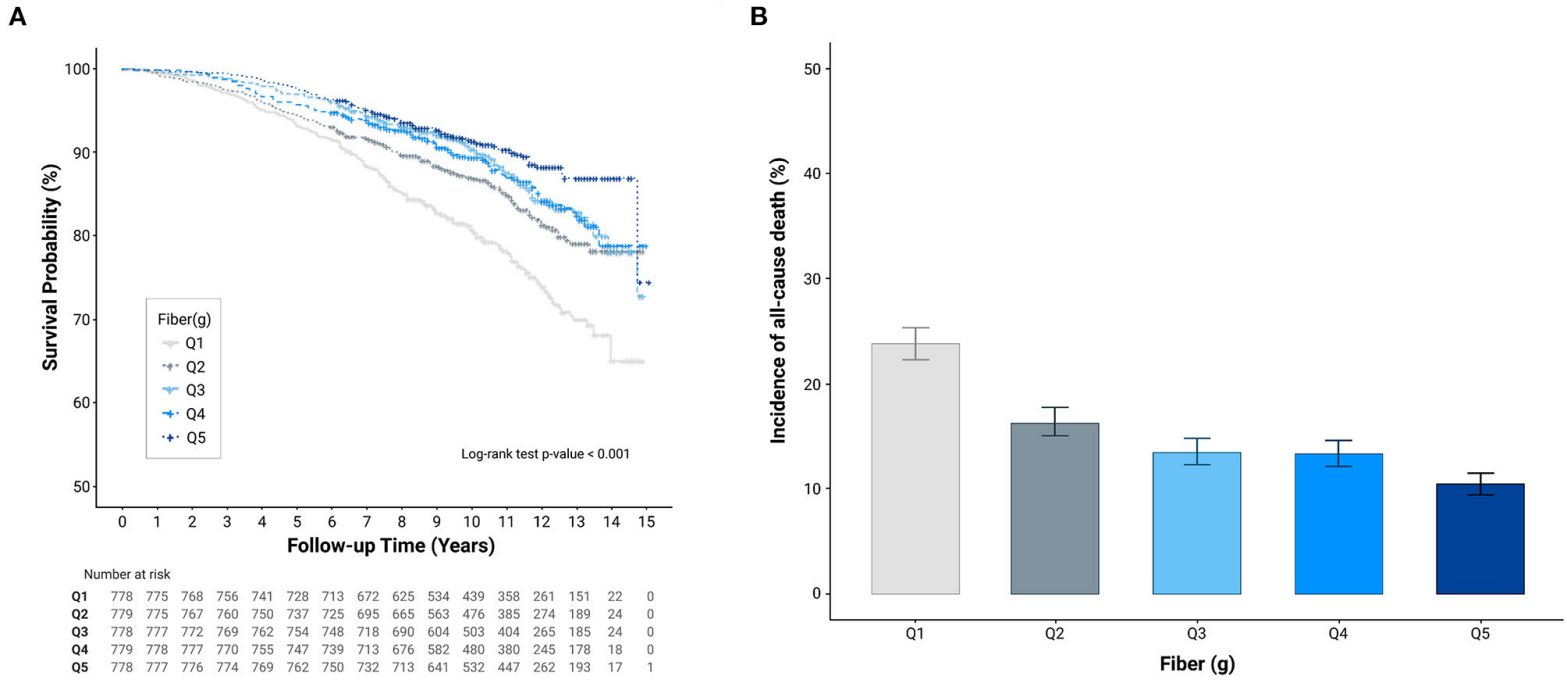
Cumulative incidence of all-cause mortality according to quintiles of dietary fiber intake (g/day). **(A)** Kaplan-Meier curves **(B)** All-cause mortality incidence rates.

**Figure 2:**
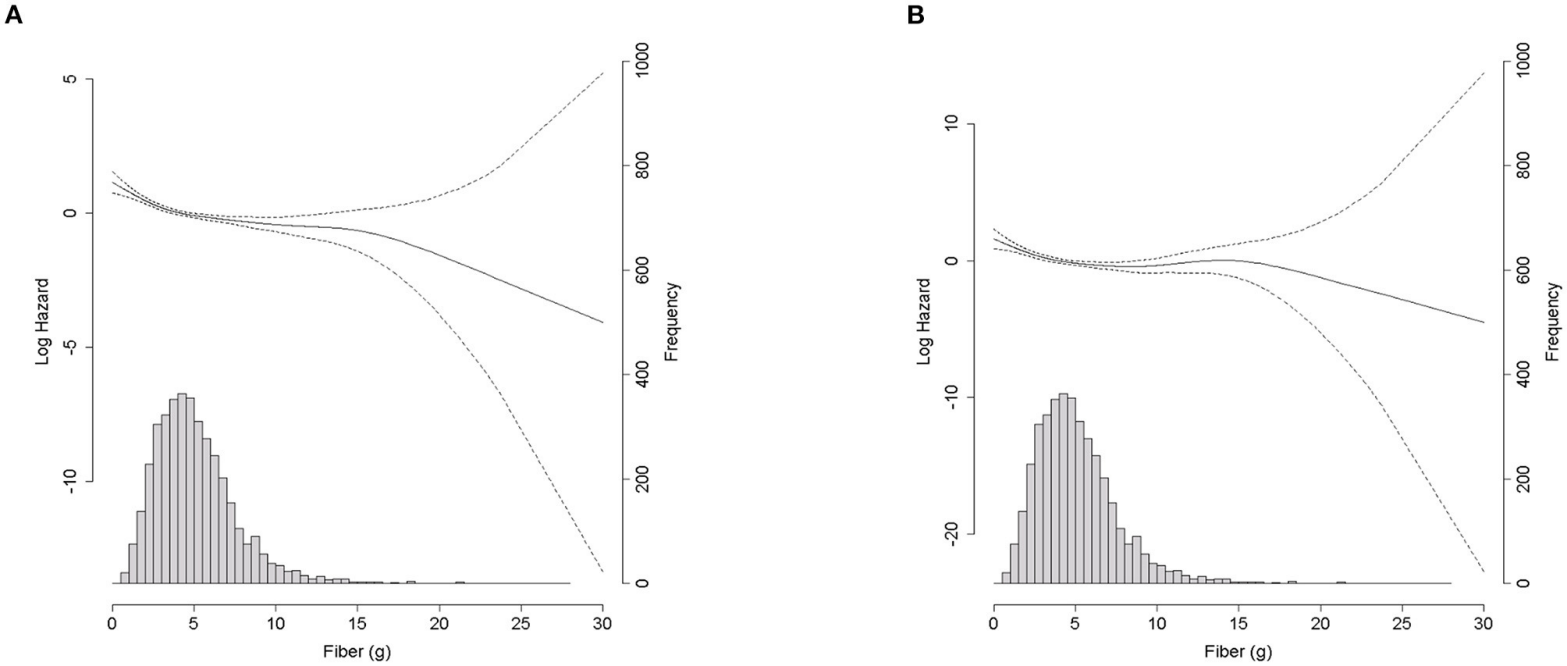
Density plot of fiber intake (g/day) and spline curve for all-cause mortality and CVD mortality according to dietary fiber intake (g/day). **(A)** All-cause mortality. **(B)** Cardiovascular motality.

As shown in Figure 1A, Kaplan–Meier curves revealed a significantly lower cumulative incidence of total mortality in Q5, followed by that from Q4 to Q1 (log-rank test *P* < 0.001). Figure 1B shows the all-cause mortality incidence rates (per 1,000 person-years) and their 95% CIs. Participants with higher fiber intakes had a lower incidence per 1,000 person years. Cox proportional hazards spline curves showed inverse association between dietary fiber intake and total mortality in patients with CKD (Figure 2A). There were similar inverse association between dietary fiber intake and CVD mortality in patients with CKD (Figure 2B).

The HRs and 95% CIs for all-cause mortality across the dietary fiber intake quintiles are presented in Table 2. Compared to Q1, the HRs (95% CI) for all-cause mortality in Q2, Q3, Q4, and Q5 were 0.71 (0.57–0.90), 0.66 (0.52–0.84), 0.69 (0.54–0.88), and 0.55 (0.42–0.72), respectively, after adjusting for age, sex, and BMI. In the isocaloric model, compared to Q1, the HRs (95% CI) for all-cause mortality in Q2, Q3, Q4, and Q5 were 0.75 (0.65–0.95), 0.72 (0.55–0.93), 0.75 (0.57–0.98), and 0.61 (0.45–0.85), respectively, after adjusting for age, sex, BMI, smoking, alcohol consumption, exercise, and total calorie intake. After additionally adjusting for history of hypertension, diabetes, dyslipidemia, and baseline eGFR, a significant inverse association between dietary fiber intake and all-cause mortality was noted in patients with CKD [Q5 vs. Q1; 0.67 (0.48–0.93), *P* = 0.016].

**Table 2:** Multiple cox proportional hazard regression analysis of dietary fiber intake quintiles for all-cause mortality and cardiovascular mortality.

	**Model 1**		**Model 2**		**Model 3**		**Model 4**	
**Variables**	**HR (95% CI)**	***P*** **-value**	**HR (95% CI)**	***P*** **-value**	**HR (95% CI)**	***P*** **-value**	**HR (95% CI)**	***P*** **-value**
**All-cause mortality**								
**Dietary fiber intake (g/day)**								
Q1 (0.50, 3.01)	Ref		Ref		Ref		Ref	
Q2 (3.02, 4.15)	0.71 (0.57–0.90)	0.004	0.75 (0.60–0.95)	0.016	0.75 (0.60–0.95)	0.018	0.78 (0.62–0.98)	0.035
Q3 (4.16, 5.26)	0.66 (0.52–0.84)	0.001	0.72 (0.55–0.93)	0.011	0.71 (0.55–0.91)	0.008	0.74 (0.57–0.96)	0.022
Q4 (5.27, 6.76)	0.69 (0.54–0.88)	0.003	0.75 (0.57–0.98)	0.035	0.75 (0.57–0.99)	0.040	0.76 (0.58–1.00)	0.051
Q5 (6.77, 27.6)	0.55 (0.42–0.72)	<0.001	0.61 (0.45–0.85)	0.003	0.63 (0.46–0.87)	0.005	0.67 (0.48–0.93)	0.016
**CVD mortality**								
**Dietary fiber intake (g/day)**								
Q1 (0.50, 3.01)	Ref		Ref		Ref		Ref	
Q2 (3.02, 4.15)	0.51 (0.32–0.81)	0.005	0.53 (0.33–0.86)	0.011	0.54 (0.33–0.87)	0.012	0.53 (0.33–0.87)	0.011
Q3 (4.16, 5.26)	0.68 (0.42–1.08)	0.098	0.72 (0.44–1.18)	0.194	0.72 (0.44–1.18)	0.189	0.73 (0.45–1.20)	0.213
Q4 (5.27, 6.76)	0.51 (0.31–0.86)	0.011	0.55 (0.31–0.97)	0.038	0.55 (0.31–0.96)	0.037	0.55 (0.31–0.97)	0.038
Q5 (6.77, 27.6)	0.50 (0.29–0.86)	0.012	0.56 (0.29–1.07)	0.077	0.56 (0.29–1.08)	0.082	0.58 (0.30–1.11)	0.099

*Model 1: adjusted for age, sex, and BMI*.

*Model 2: adjusted for age, sex, BMI, smoking, alcohol intake, exercise, and total calorie intake*.

*Model 3: adjusted for age, sex, BMI, smoking, alcohol intake, exercise, total calorie intake, hypertension, diabetes, and dyslipidemia*.

*Model 4: adjusted for age, sex, BMI, smoking, alcohol intake, exercise, total calorie intake, hypertension, diabetes, dyslipidemia, and baseline eGFR*.

Additionally, we examined the association between dietary fiber intake and CVD mortality in patients with CKD. There were 149 CVD death events during the follow-up period (Supplementary Table 3).

Compared to Q1, the HR and 95% CI for CVD mortality in Q5 was 0.50 (0.29–0.86) after adjusting for age, sex, and BMI (*P* = 0.012). Compared to Q1, the HR and 95% CI for CVD mortality in Q5 was 0.58 (0.30–1.11) after adjusting for age, sex, BMI, smoking, alcohol consumption, exercise, total calorie intake, hypertension, diabetes, dyslipidemia, and baseline eGFR (*P* = 0.099).

Each 1 g/day increase of total dietary fiber was associated with HR 0.92, 95% CI 0.88–0.95 for all-cause mortality and HR 0.91, 95% CI (0.84–0.99) for CVD mortality. With further adjustment for age, sex, BMI, smoking, alcohol consumption, exercise, and total calorie intake, hypertension, diabetes, dyslipidemia, and eGFR, the corresponding HR and 95% CI for all-cause mortality and CVD mortality were 0.94, 0.90–0.99 (*P* = 0.014) and 0.93, (0.84–1.03) (*P* = 0.161), respectively (data not shown).

We also examined the association between total fiber intake and all-cause mortality in patients with CKD according to the CKD stages, sex, BMI criteria 25kg/m^2^ (Supplementary Table 4). The numbers (%) of CKD 3, 4, and 5 were 3,717 (95.5%), 122 (3.1%), and 53 (1.4%), respectively. There were significant association between dietary fiber intake and all-cause mortality in the only CKD stage 3, female, and BMI <25 kg/m^2^.

## Discussion

Despite the accumulating evidence on the benefits of dietary fiber in the general population, there is a lack of representative data on mortality in patients with CKD, especially in East Asia. In this study, we observed a significant and inverse relationship between dietary fiber intake and mortality risk in patients with CKD in a Korean prospective cohort followed up for 10 years. A similar trend was noted in the association between dietary fiber intake and CVD mortality.

The American Dietetic Association recommended a daily dietary fiber intake of 25–30 g for adults in the general population (32). Similarly, the 2015 Dietary Reference Intake for Koreans (KDRI) suggested that the sufficient daily fiber intake for Korean adults is 25 g/day for men and 20 g/day for women, regardless of age (33). However, to our knowledge, there are no specific guidelines for dietary fiber intake in the CKD population in the available nephrology guidelines.

Dietary recommendations for patients with CKD are different from those for healthy people. Patients with advanced CKD are frequently advised to restrict the intake of vegetables, fruits, legumes, nuts, and whole-grains due to their high potassium content and the risk of hyperkalemia and hyperphosphatemia (34). Therefore, fiber intake in patients with CKD is generally lower than that in the general population (8). Data from the National Health and Nutrition Examination Survey (NHANES) in the US showed that total dietary fiber intake in the CKD population averaged 15.4 g/day, which is far below the recommended 25–30 g/day intake for the general population (8). In this study, the average daily fiber intake of patients with CKD was only 5.1 g/day, which was extremely low. This result may be due to the competing dietary potassium and phosphorus concerns. However, a high-fiber diet increases fecal potassium excretion by increasing stool volume (35), and the bioavailability of potassium and phosphorus in high-fiber foods is lower than that in other phosphorus and potassium foods, especially processed foods (1). Supporting this, there were weak or no correlations between dietary potassium and serum potassium in advanced CKD (36, 37), and cohort studies fail to prove a close relationship between diets rich in fibers and serum potassium levels (38). Considering these results, the recent Kidney Disease Outcomes Quality Initiative (KDOQI) guidelines recommended “adjusting” potassium intake “when needed” to maintain potassium levels in the normal range, but not routinely, as this may result in deficiencies in other healthy nutrients, such as fiber (39). Interestingly, low potassium and phosphorus intake was associated with increased odds of advanced stage CKD in Korean hypertensive patients (40). In this study, we observed that higher dietary fiber intake was associated with a reduced risk of death in patients with CKD. When comparing persons with dietary fiber intakes in the top and bottom quintiles, we found a statistically significant inverse association between fiber intake and all-cause mortality, with an overall relative risk of 0.63 (95% CI: 0.46–0.87). Together, these studies suggest that, regardless of the risk of hyperkalemia and hyperphosphatemia, a higher fiber intake may be beneficial for Koreans with CKD. However, there has been economic growth and lifestyle changes in Korea over the past decades. Consequently, there has been a noticeable shift in nutrient intake from grain-based foods to western style meals focused on protein and lipid (41). Although rice consumption still constitutes the largest proportion, it has continuously declined to one third of total energy intake. This is much lower than the intake of other Asians, whose trends are opposite to the current situation in Western countries where the consumption of rice is increasing (41). A national dietary plan and strategy should be set to establish nutritional transition toward higher fiber intake for Koreans with CKD.

Several mechanisms support the inverse association between dietary fiber intake and mortality in patients with CKD. The bulking effect of dietary fiber is important for colonic health and gut motility. Dietary fiber leads to uremic toxin excretion by increasing stool output (42), secondary to increased proteolytic activities by protein fermenting bacteria (43). A systemic review including 203 patients with CKD from seven reports showed that dietary fiber reduced p-cresyl sulfate, thereby protecting the intestinal epithelial barrier of patients with CKD (44). Favorable alteration in the gut microbiome not only modulates uremic toxins (45) but also chronic inflammatory pathways (11). Compared with the healthy population, patients with CKD exhibited increased serum levels of CRP, IL-6, interferon-γ, and tumor necrosis factor-α (46). However, increased dietary fiber intake and supplementation were associated with a decreased systemic inflammatory state, including the C-reactive protein level (8, 47). Because uremic toxins and chronic inflammation are central to the progression of CKD, some have hypothesized that fiber may slow the progression of CKD. Emerging studies have shown that high dietary fiber intake is associated with the primary prevention of CKD and delaying of CKD progression (48, 49). The Tehran Lipid and Glucose Study, including 1 630 participants with 6.1 years of follow-up, showed an 11% lower risk of incident CKD per 5-g/d increase in total fiber intake (48). In this study, compared to the lowest tertile groups, the highest tertile vegetable fiber and legume fiber groups were significantly associated with lower risk of CKD incidence (48). In the Blue Mountains Eye Study, after 5 years of follow-up, high cereal fiber intake was associated with a 50% reduction in the incidence of CKD (49). A meta-analysis of 14 controlled feeding trials including patients with CKD demonstrated that dietary fiber can reduce serum urea and creatinine levels with a dose dependent response for serum creatinine (1). No specific recommendations have been made in relation to the preferred types of dietary fiber consumption, although it is recommended that ≥50% of all grains consumed should be whole grains (50). Although we could not distinguish fiber types, the main sources of fiber intake in Koreans are grains and vegetables (51).

Dietary fiber intake may modify the cardio-metabolic risk profile by lowering blood pressure (52) and LDL-C levels (53), ameliorating postprandial hyperglycemia, and enhancing peripheral insulin sensitivity (54), which may contribute to complications and mortality related to CVD. Based on the available published literature, there appear to be an association between dietary fiber intake and mortality from CVD, including coronary heart disease and cerebrovascular disease (55, 56). Threapleton et al. published a systematic review and meta-analysis including 22 prospective cohort studies reporting on inverse associations between dietary fiber intake and coronary heart disease or CVD (55). A dose–response meta-analysis from 15 prospective cohort studies also revealed a pooled relative risk reduction of 9% in CVD mortality from a dietary fiber intake increment of 10 g/day (56). Of great importance is the fact that the leading cause of death in patients with CKD is CVD rather than progression to end-stage renal disease (57). In line with previous studies, we observed a borderline statistical significance in the association between dietary fiber intake and CVD mortality, with 149 CVD death events in patients with CKD. Further prospective studies including larger numbers of deaths would be needed to confirm the association between dietary fiber intake and CVD mortality in Korean patients with CKD.

In the current study, non-significant associations was noted between dietary fiber intake and all-cause mortality in CKD stage 4 and 5, men and participants with BMI ≥ 25 kg/m^2^. There were very few patients with CKD stages 4 and 5 in the dataset. A small dataset could lead to overfitting problems in logistic regression models. Although the exact reasons for the different association between dietary fiber intake and mortality in men and overweight individuals are unclear, dietary fiber sources might lead to different results. Further research is needed on the relationship between fiber intake and mortality according to sex and obesity.

Our study has several limitations. First, foods containing high fiber are commonly plant-based foods, such as vegetables, fruits, nuts, and seeds. These foods contain not only dietary fiber but also various vitamins, minerals, and polyphenols. Therefore, it is unclear whether the dietary fiber itself had beneficial effects or whether other nutrients in the food containing dietary fiber had positive effects on mortality in patients with CKD. Therefore, caution is needed in interpreting these results. Second, we could not obtain detailed information on the dietary fiber sources. In future study, we will attempt to investigate the association between fiber type and mortality in CKD patients. Third, previous studies using the same data (KoGES) showed a low dietary fiber intake (mean 6.3 ± 3.6 g/day) in the general middle-aged Korean adult population (30, 58). However, the mean average dietary fiber intake among patients with CKD was too low compared with the recommended dietary fiber intake for Koreans (59). Therefore, it is unclear whether our results could be generalized to other countries with different food habits. Forth, we did not consider the effects of changes of fiber intake during the follow-up period. The dataset is only composed of baseline survey data and data linkage with national mortality data. The consideration of changes in dietary fiber intake during the follow-up period will be incorporated into future study. Finally, we could not obtain information on urine albumin and creatinine levels or dialysis status.

Despite these limitations, our study has several strengths. The present study provided data based on a validated 103-question FFQ in a population-based sample and had a rather long follow-up duration. In addition, this is the first study to examine the association between dietary fiber intake and all-cause mortality in a Korean population with CKD.

## Conclusions

In this study, dietary fiber intake was associated with a lower risk of all-cause mortality in patients with CKD. The average daily fiber intake of Korean patients with CKD was extremely low; however, we found that small increments in the fiber intake reduced the risk of premature death by 37% and had desirable effects on cardiovascular mortality. This finding highlights the need for encouraging adequate fiber intake through consumption of whole grains, vegetables, and legumes in CKD patients. Further study is needed to define the recommended fiber intake in patients with CKD and the health impacts of different types of fiber on CKD related complications and mortality.

## Data Availability Statement

Publicly available datasets were analyzed in this study. This data can be found here: https://www.kdca.go.kr/contents.es?mid=a40504010000.

## Ethics Statement

The studies involving human participants were reviewed and approved by the Institutional Review Boards of Yongin Severance Hospital (IRB Number: 9-2021-0066). The patients/participants provided their written informed consent to participate in this study.

## Author Contributions

Y-JK, HL, GP, and J-WL contributed to the conception or design of the work, contributed to the acquisition, analysis, or interpretation of the data, and drafting of the manuscript. All authors critically revised the manuscript, provided final approval and agree to be accountable for all aspects of the work, and ensuring integrity and accuracy.

## Funding

This work was supported by the Korea Institute of Planning and Evaluation for Technology in Food, Agriculture and Forestry (IPET) through High Value-Added Food Technology Development Program funded by the Ministry of Agriculture, Food and Rural Affairs (MAFRA) (321030051HD030). The grant was awarded to J-WL and Y-JK.

## Conflict of Interest

The authors declare that the research was conducted in the absence of any commercial or financial relationships that could be construed as a potential conflict of interest.

## Publisher's Note

All claims expressed in this article are solely those of the authors and do not necessarily represent those of their affiliated organizations, or those of the publisher, the editors and the reviewers. Any product that may be evaluated in this article, or claim that may be made by its manufacturer, is not guaranteed or endorsed by the publisher.
