# Supplementary material for: Association Between Dietary Fiber Intake and All-Cause and Cardiovascular Mortality in Middle Aged and Elderly Adults With Chronic Kidney Disease
Source: Front Nutr. 2022 Apr 19;9:863391. doi: 10.3389/fnut.2022.863391 (PMC9062480; doi:10.3389/fnut.2022.863391)
Supplement: Supplementary file 1 [file Data_Sheet_1.docx]

**SUPPLEMENTARY MATERIAL**

**Supplementary Figure S1. Flow chart of study population**

**Selected participants**

(*n* = 143 050)

**Participants from Korean Genome and Epidemiology Study**

(*n* = 211 571)

**Mortality**

(*n* = 602)

**Exclusion criteria**

1) Missing lifestyle data (*n* = 2 231)

2) Missing laboratory test data (*n* = 5 853)

3) Implausible daily total calorie intake (< 500 or > 6 000 kcal/day) and missing nutrition intake data (*n* = 14 007)

4) Missing mortality data (*n* = 54 530)

5) Those who died in enrolled year (*n* = 63)

**Participants with CKD**

(*n* = 3 892)

**Supplementary Table S1. Baseline characteristics of the cohort according to mortality status**

| **Characteristics** | **Alive** | **All-cause mortality** | ***P*-value** |
| --- | --- | --- | --- |
| N | 3290 | 602 |  |
| Gender (men) | 1209 (36.75) | 286 (47.51) | < 0.0001 |
| Age (years) | 61.829±8.011 | 68.527±7.307 | < 0.0001 |
| BMI (kg/m^2^) | 24.928±3.004 | 24.220±3.222 | < 0.0001 |
| Waist circumference (cm) | 85.004±8.764 | 85.852±9.310 | 0.0388 |
| Systolic BP (mmHg) | 127.131±17.012 | 130.153±19.931 | 0.0005 |
| Diastolic BP (mmHg) | 77.827±10.275 | 76.442±10.898 | 0.0026 |
| Laboratory |  |  |  |
| Glucose (mg/dl) | 102.617±33.150 | 112.090±45.675 | < 0.0001 |
| HbA1c (%) | 6.109±1.080 | 6.790±1.711 | < 0.0001 |
| Total cholesterol (mg/dl) | 198.374±39.604 | 193.141±40.793 | 0.003 |
| HDL-C (mg/dl) | 46.718±12.036 | 43.419±11.521 | < 0.0001 |
| LDL-C (mg/dl) | 121.111±35.998 | 117.593±35.523 | 0.0272 |
| Triglycerides (mg/dl) | 153.851±95.548 | 160.650±91.055 | 0.106 |
| BUN | 19.954±8.221 | 22.311±10.164 | < 0.0001 |
| Creatinine | 1.329±0.774 | 1.512±1.049 | < 0.0001 |
| eGFR (mL/min/1.73 m^2^) | 52.706±9.125 | 49.366±11.619 | < 0.0001 |
| AST (IU/L) | 25.307±10.071 | 26.457±11.034 | 0.0177 |
| ALT (IU/L) | 22.991±13.593 | 22.607±12.925 | 0.5223 |
| Smoking status |  |  | < 0.0001 |
| Never smoker | 2378 (72.28) | 361 (59.97) |  |
| Former smoker | 641 (19.48) | 147 (24.42) |  |
| Current smoker | 271 (8.24) | 94 (15.61) |  |
| Alcohol intake |  |  | < 0.0001 |
| Never drinker | 1999 (60.76) | 322 (53.49) |  |
| Former drinker | 226 (6.87) | 86 (14.29) |  |
| Current drinker | 1065 (32.37) | 194 (32.23) |  |
| Regular exercise^,^ n (%) | 1542 (46.8) | 205 (34.1) | < 0.0001 |
| Hypertension | 881 (26.78) | 191 (31.73) | 0.0124 |
| Diabetes mellitus | 496 (15.08) | 140 (23.26) | < 0.0001 |
| Dyslipidemia | 2083 (63.31) | 372 (61.79) | 0.4777 |
| Residential area |  |  | < 0.0001 |
| Urban, n (%) | 2102 (63.89) | 275 (45.68) |  |
| Rural, n (%) | 1188 (36.11) | 327 (54.32) |  |
| Total energy intake (kcal/day) | 1603.562±502.853 | 1479.197±458.709 | < 0.0001 |
| Carbohydrate intake (g/day) | 294.558±84.589 | 276.089±79.464 | < 0.0001 |
| Carbohydrate (%) | 74.193±6.707 | 75.396±6.784 | < 0.0001 |
| Fat intake (g/day) | 22.179±15.036 | 18.743±13.239 | < 0.0001 |
| Fat (%) | 11.838±5.291 | 10.763±5.403 | < 0.0001 |
| Protein intake (g/day) | 51.737±22.167 | 45.869±19.448 | < 0.0001 |
| Protein (%) | 12.719±2.512 | 12.205±2.553 | < 0.0001 |
| Ca, mg | 392.415±245.304 | 324.101±208.410 | < 0.0001 |
| P, mg | 797.000±321.109 | 705.647±279.657 | < 0.0001 |
| Fe, mg | 8.697±4.297 | 7.453±3.878 | < 0.0001 |
| Vit. A, RE | 411.698±310.127 | 330.603±247.519 | < 0.0001 |
| Vit. B1, mg | 0.868±0.375 | 0.760±0.351 | < 0.0001 |
| Vit. B2, mg | 0.776±0.408 | 0.648±0.337 | < 0.0001 |
| Niacin, mg | 12.610±5.378 | 11.125±4.830 | < 0.0001 |
| Vit. C, mg | 93.572±61.895 | 73.058±50.169 | < 0.0001 |
| Zinc, ug | 7.095±3.177 | 6.366±3.507 | < 0.0001 |
| Vit. B6, mg | 1.414±0.611 | 1.233±0.545 | < 0.0001 |
| Folate, ug | 191.637±108.264 | 161.556±93.621 | < 0.0001 |
| Retinol, ug | 53.982±55.590 | 41.745±42.372 | < 0.0001 |
| Carotene, ug | 2088.238±1676.493 | 1687.032±1324.942 | < 0.0001 |
| Fiber, g | 5.230±2.597 | 4.434±2.287 | < 0.0001 |
| Vit. E, mg | 6.999±3.957 | 5.868±3.354 | < 0.0001 |

BMI, body mass index; WC, waist circumference; SBP, systolic blood pressure; DBP, diastolic blood pressure; FBG, fasting blood glucose; TC, total cholesterol; HDL-C, high density lipoprotein cholesterol; LDL-C, low density lipoprotein cholesterol; TG, triglyceride; BUN, blood urea nitrogen; Cr, creatinine; AST, aspartate transaminase; ALT, alanine transaminase; HTN, hypertension; DM, diabetes mellitus; Vit, vitamin; RE, retinol equivalents

**Supplementary Table S2. Post-hoc analysis results from the comparison of the study population**

| **Characteristics** | **1 vs 2** | **1 vs 3** | **1 vs 4** | **1 vs 5** | **2 vs 3** | **2 vs 4** | **2 vs 5** | **3 vs 4** | **3 vs 5** | **4 vs 5** |
| --- | --- | --- | --- | --- | --- | --- | --- | --- | --- | --- |
| Gender (men) | 0.0042 | 0.0047 | < 0.0001 | < 0.0001 | 0.9741 | 0.0497 | 0.0533 | 0.0461 | 0.0495 | 0.9766 |
| Age (years) | 0.0007 | < 0.0001 | < 0.0001 | < 0.0001 | 0.0019 | 0.0003 | < 0.0001 | 0.5825 | 0.0149 | 0.0592 |
| BMI (kg/m^2^) | 0.1586 | 0.012 | 0.0018 | 0.0011 | 0.2699 | 0.0857 | 0.0639 | 0.5385 | 0.4531 | 0.8921 |
| Waist circumference (cm) | 0.0242 | 0.0288 | 0.0001 | 0.0027 | 0.9456 | 0.1062 | 0.457 | 0.0922 | 0.4168 | 0.383 |
| Systolic BP (mmHg) | 0.9969 | 0.623 | 0.4825 | 0.5827 | 0.6257 | 0.4848 | 0.5799 | 0.8332 | 0.2979 | 0.2106 |
| Diastolic BP (mmHg) | 0.4152 | 0.4933 | 0.2436 | 0.0072 | 0.8969 | 0.7252 | 0.0608 | 0.6306 | 0.0451 | 0.1276 |
| Laboratory |  |  |  |  |  |  |  |  |  |  |
| Glucose (mg/dl) | 0.7297 | 0.6726 | 0.1431 | 0.0242 | 0.4423 | 0.0703 | 0.0093 | 0.2976 | 0.0671 | 0.4292 |
| HbA1c (%) | 0.3638 | 0.2579 | 0.0905 | 0.1615 | 0.8321 | 0.4314 | 0.6225 | 0.5575 | 0.7739 | 0.7677 |
| Total cholesterol (mg/dl) | 0.4023 | 0.5131 | 0.5944 | 0.2167 | 0.8545 | 0.7602 | 0.6904 | 0.9031 | 0.5609 | 0.4818 |
| HDL-C (mg/dl) | 0.2087 | 0.0043 | 0.0074 | 0.0213 | 0.1095 | 0.155 | 0.2949 | 0.8579 | 0.5801 | 0.7081 |
| LDL-C (mg/dl) | 0.2337 | 0.535 | 0.8758 | 0.149 | 0.5685 | 0.1779 | 0.8004 | 0.4374 | 0.4107 | 0.1098 |
| Triglycerides (mg/dl) | 0.2394 | 0.1781 | 0.9628 | 0.221 | 0.8647 | 0.2584 | 0.9621 | 0.1934 | 0.9022 | 0.2389 |
| BUN | 0.2063 | 0.1638 | 0.2905 | 0.0677 | 0.8973 | 0.836 | 0.5729 | 0.7368 | 0.6639 | 0.4409 |
| Creatinine | 0.1109 | 0.1298 | 0.5023 | 0.0991 | 0.9371 | 0.3555 | 0.9558 | 0.3982 | 0.8932 | 0.3275 |
| eGFR (mL/min/1.73 m2) | 0.0176 | 0.004 | 0.0121 | < 0.0001 | 0.6153 | 0.892 | 0.0123 | 0.7138 | 0.0456 | 0.018 |
| AST (IU/L) | 0.4791 | 0.9619 | 0.6182 | 0.588 | 0.4498 | 0.8343 | 0.8681 | 0.5848 | 0.5554 | 0.9656 |
| ALT (IU/L) | 0.2896 | 0.1379 | 0.0282 | 0.0074 | 0.6707 | 0.2552 | 0.1049 | 0.4756 | 0.2316 | 0.6301 |
| Smoking status | 0.052 | 0.0001 | 0.0016 | 0.0084 | 0.1845 | 0.1132 | 0.5765 | 0.0432 | 0.2433 | 0.5429 |
| Alcohol intake | 0.0424 | 0.0019 | 0.0002 | 0.0001 | 0.2765 | 0.2669 | 0.04 | 0.4349 | 0.6418 | 0.1595 |
| Regular exercise^,^ | < 0.0001 | < 0.0001 | < 0.0001 | < 0.0001 | 0.1195 | 0.0219 | 0.0002 | 0.4614 | 0.0274 | 0.1412 |
| Hypertension | 0.4804 | 0.9093 | 0.9216 | 0.1613 | 0.4125 | 0.4213 | 0.0352 | 0.9876 | 0.1981 | 0.1926 |
| Diabetes mellitus | 0.9027 | 0.8935 | 0.5328 | 0.0415 | 0.9907 | 0.4555 | 0.0307 | 0.4486 | 0.0298 | 0.1562 |
| Dyslipidemia | 0.3886 | 0.8342 | 0.6602 | 0.9166 | 0.284 | 0.6725 | 0.3336 | 0.5164 | 0.9167 | 0.5862 |
| Residential area | < 0.0001 | < 0.0001 | < 0.0001 | < 0.0001 | 0.016 | < 0.0001 | < 0.0001 | 0.0134 | 0.0005 | 0.3017 |
| Total energy intake (kcal/day) | < 0.0001 | < 0.0001 | < 0.0001 | < 0.0001 | < 0.0001 | < 0.0001 | < 0.0001 | < 0.0001 | < 0.0001 | < 0.0001 |
| Carbohydrate intake (g/day) | < 0.0001 | < 0.0001 | < 0.0001 | < 0.0001 | < 0.0001 | < 0.0001 | < 0.0001 | < 0.0001 | < 0.0001 | < 0.0001 |
| Carbohydrate (%) | < 0.0001 | < 0.0001 | < 0.0001 | < 0.0001 | < 0.0001 | < 0.0001 | < 0.0001 | < 0.0001 | < 0.0001 | < 0.0001 |
| Fat intake (g/day) | < 0.0001 | < 0.0001 | < 0.0001 | < 0.0001 | < 0.0001 | < 0.0001 | < 0.0001 | < 0.0001 | < 0.0001 | < 0.0001 |
| Fat (%) | < 0.0001 | < 0.0001 | < 0.0001 | < 0.0001 | < 0.0001 | < 0.0001 | < 0.0001 | < 0.0001 | < 0.0001 | 0.0001 |
| Protein intake (g/day) | < 0.0001 | < 0.0001 | < 0.0001 | < 0.0001 | < 0.0001 | < 0.0001 | < 0.0001 | < 0.0001 | < 0.0001 | < 0.0001 |
| Protein (%) | < 0.0001 | < 0.0001 | < 0.0001 | < 0.0001 | < 0.0001 | < 0.0001 | < 0.0001 | < 0.0001 | < 0.0001 | < 0.0001 |
| Ca, mg | < 0.0001 | < 0.0001 | < 0.0001 | < 0.0001 | < 0.0001 | < 0.0001 | < 0.0001 | < 0.0001 | < 0.0001 | < 0.0001 |
| P, mg | < 0.0001 | < 0.0001 | < 0.0001 | < 0.0001 | < 0.0001 | < 0.0001 | < 0.0001 | < 0.0001 | < 0.0001 | < 0.0001 |
| Fe, mg | < 0.0001 | < 0.0001 | < 0.0001 | < 0.0001 | < 0.0001 | < 0.0001 | < 0.0001 | < 0.0001 | < 0.0001 | < 0.0001 |
| Vit. A, RE | < 0.0001 | < 0.0001 | < 0.0001 | < 0.0001 | < 0.0001 | < 0.0001 | < 0.0001 | < 0.0001 | < 0.0001 | < 0.0001 |
| Vit. B1, mg | < 0.0001 | < 0.0001 | < 0.0001 | < 0.0001 | < 0.0001 | < 0.0001 | < 0.0001 | < 0.0001 | < 0.0001 | < 0.0001 |
| Vit. B2, mg | < 0.0001 | < 0.0001 | < 0.0001 | < 0.0001 | < 0.0001 | < 0.0001 | < 0.0001 | < 0.0001 | < 0.0001 | < 0.0001 |
| Niacin, mg | < 0.0001 | < 0.0001 | < 0.0001 | < 0.0001 | < 0.0001 | < 0.0001 | < 0.0001 | < 0.0001 | < 0.0001 | < 0.0001 |
| Vit. C, mg | < 0.0001 | < 0.0001 | < 0.0001 | < 0.0001 | < 0.0001 | < 0.0001 | < 0.0001 | < 0.0001 | < 0.0001 | < 0.0001 |
| Zinc, ug | < 0.0001 | < 0.0001 | < 0.0001 | < 0.0001 | < 0.0001 | < 0.0001 | < 0.0001 | < 0.0001 | < 0.0001 | < 0.0001 |
| Vit. B6, mg | < 0.0001 | < 0.0001 | < 0.0001 | < 0.0001 | < 0.0001 | < 0.0001 | < 0.0001 | < 0.0001 | < 0.0001 | < 0.0001 |
| Folate, ug | < 0.0001 | < 0.0001 | < 0.0001 | < 0.0001 | < 0.0001 | < 0.0001 | < 0.0001 | < 0.0001 | < 0.0001 | < 0.0001 |
| Retinol, ug | 0.0002 | < 0.0001 | < 0.0001 | < 0.0001 | < 0.0001 | < 0.0001 | < 0.0001 | < 0.0001 | < 0.0001 | < 0.0001 |
| Carotene, ug | < 0.0001 | < 0.0001 | < 0.0001 | < 0.0001 | < 0.0001 | < 0.0001 | < 0.0001 | < 0.0001 | < 0.0001 | < 0.0001 |
| Vit. E, mg | < 0.0001 | < 0.0001 | < 0.0001 | < 0.0001 | < 0.0001 | < 0.0001 | < 0.0001 | < 0.0001 | < 0.0001 | < 0.0001 |

BMI, body mass index; WC, waist circumference; SBP, systolic blood pressure; DBP, diastolic blood pressure; FBG, fasting blood glucose; TC, total cholesterol; HDL-C, high density lipoprotein cholesterol; LDL-C, low density lipoprotein cholesterol; TG, triglyceride; BUN, blood urea nitrogen; Cr, creatinine; AST, aspartate transaminase; ALT, alanine transaminase; Vit, vitamin; RE, retinol equivalents

**Supplementary Table S3. Baseline characteristics of the cohort according to CVD mortality status**

| **Characteristics** | **Alive** | **CVD mortality** | **p-value** |
| --- | --- | --- | --- |
| N | 3290 | 149 |  |
| Gender (men) | 1209 (36.75) | 59 (39.60) | 0.4807 |
| Age (years) | 61.829±8.011 | 69.195±7.225 | < 0.0001 |
| BMI (kg/m^2^) | 24.928±3.004 | 24.185±3.242 | 0.0033 |
| Waist circumference (cm) | 85.004±8.764 | 85.846±8.945 | 0.2521 |
| Systolic BP (mmHg) | 127.131±17.012 | 132.282±21.940 | 0.0053 |
| Diastolic BP (mmHg) | 77.827±10.275 | 76.211±12.407 | 0.1196 |
| Laboratory |  |  |  |
| Glucose (mg/dl) | 102.617±33.150 | 112.550±43.344 | 0.0065 |
| HbA1c (%) | 6.109±1.080 | 7.030±1.641 | 0.0074 |
| Total cholesterol (mg/dl) | 198.374±39.604 | 192.852±39.779 | 0.0961 |
| HDL-C (mg/dl) | 46.718±12.036 | 43.953±12.011 | 0.0061 |
| LDL-C (mg/dl) | 121.111±35.998 | 117.639±35.215 | 0.2491 |
| Triglycerides (mg/dl) | 153.851±95.548 | 156.302±92.772 | 0.7591 |
| BUN | 19.954±8.221 | 21.885±8.729 | 0.0052 |
| Creatinine | 1.329±0.774 | 1.429±1.023 | 0.2414 |
| eGFR | 52.706±9.125 | 50.496±10.709 | 0.0142 |
| AST (IU/L) | 25.307±10.071 | 26.859±11.104 | 0.0672 |
| ALT (IU/L) | 22.991±13.593 | 22.678±13.125 | 0.7831 |
| Smoking status |  |  | 0.0144 |
| Never smoker | 2378 (72.28) | 96 (64.43) |  |
| Former smoker | 641 (19.48) | 31 (20.81) |  |
| Current smoker | 271 (8.24) | 22 (14.77) |  |
| Alcohol intake |  |  | 0.0079 |
| Never drinker | 1999 (60.76) | 88 (59.06) |  |
| Former drinker | 226 (6.87) | 20 (13.42) |  |
| Current drinker | 1065 (32.37) | 41 (27.52) |  |
| Regular exercise, n (%) | 1542 (46.82) | 44 (29.53) | < 0.0001 |
| Hypertension | 881 (26.78) | 56 (37.58) | 0.0038 |
| Diabetes mellitus | 496 (15.08) | 38 (25.50) | 0.0006 |
| Dyslipidemia | 2083 (63.31) | 97 (65.10) | 0.6577 |
| Residential area |  |  | < 0.0001 |
| Urban, n (%) | 2102 (63.89) | 70 (46.98) |  |
| Rural, n (%) | 1188 (36.11) | 79 (53.02) |  |
| Total energy intake (kcal/day) | 1603.562±502.853 | 1432.205±457.090 | < 0.0001 |
| Carbohydrate intake (g/day) | 294.558±84.589 | 262.211±77.740 | < 0.0001 |
| Carbohydrate (%) | 74.193±6.707 | 74.263±8.502 | 0.9212 |
| Fat intake (g/day) | 22.179±15.036 | 20.142±16.004 | 0.1069 |
| Fat (%) | 11.838±5.291 | 11.792±6.782 | 0.9361 |
| Protein intake (g/day) | 51.737±22.167 | 45.638±20.800 | 0.001 |
| Protein (%) | 12.719±2.512 | 12.445±2.875 | 0.2542 |
| Ca, mg | 392.415±245.304 | 328.202±207.063 | 0.0003 |
| P, mg | 797.000±321.109 | 698.143±282.388 | < 0.0001 |
| Fe, mg | 8.697±4.297 | 7.288±3.942 | < 0.0001 |
| Vit. A, RE | 411.698±310.127 | 353.417±283.395 | 0.0244 |
| Vit. B1, mg | 0.868±0.375 | 0.772±0.405 | 0.0023 |
| Vit. B2, mg | 0.776±0.408 | 0.659±0.360 | 0.0002 |
| Niacin, mg | 12.610±5.378 | 11.171±5.300 | 0.0014 |
| Vit. C, mg | 93.572±61.895 | 70.935±50.803 | < 0.0001 |
| Zinc, ug | 7.095±3.177 | 6.329±3.196 | 0.004 |
| Vit. B6, mg | 1.414±0.611 | 1.234±0.586 | 0.0004 |
| Folate, ug | 191.637±108.264 | 163.267±106.044 | 0.0018 |
| Retinol, ug | 53.982±55.590 | 42.291±40.246 | 0.0008 |
| Carotene, ug | 2088.238±1676.493 | 1817.259±1560.452 | 0.053 |
| Fiber, g | 5.230±2.597 | 4.264±2.479 | < 0.0001 |
| Vit. E, mg | 6.999±3.957 | 5.725±3.305 | < 0.0001 |

BMI, body mass index; CVD, cardiovascular diseases; WC, waist circumference; SBP, systolic blood pressure; DBP, diastolic blood pressure; FBG, fasting blood glucose; TC, total cholesterol; HDL-C, high density lipoprotein cholesterol; LDL-C, low density lipoprotein cholesterol; TG, triglyceride; BUN, blood urea nitrogen; Cr, creatinine; AST, aspartate transaminase; ALT, alanine transaminase; HTN, hypertension; DM, diabetes mellitus; Vit, vitamin; RE, retinol equivalents

**Supplementary Table S4.** **Multiple Cox proportional hazard regression analysis of dietary fiber intake quintiles for all-cause mortality according to CKD stage, sex, and BMI criteria**

| **Subgroups** | | **Model 1** | | **Model 2** | | **Model 3** | | **Model 4** | |
| --- | --- | --- | --- | --- | --- | --- | --- | --- | --- |
|  |  | **HR(95% CI)** | **p-value** | **HR(95% CI)** | **p-value** | **HR(95% CI)** | **p-value** | **HR(95% CI)** | **p-value** |
| **CKD stage 3** | |  |  |  |  |  |  |  |  |
|  | Q1 (0.49984,3.01571) | Ref |  | Ref |  | Ref |  | Ref |  |
|  | Q2 (3.01620,4.15368) | 0.69(0.55-0.88) | 0.003 | 0.73(0.57-0.94) | 0.013 | 0.73(0.57-0.94) | 0.013 | 0.76(0.60-0.97) | 0.03 |
|  | Q3 (4.15696,5.27337) | 0.68(0.52-0.87) | 0.003 | 0.73(0.56-0.95) | 0.02 | 0.71(0.54-0.93) | 0.013 | 0.72(0.55-0.94) | 0.015 |
|  | Q4 (5.27742,6.76295) | 0.70(0.54-0.90) | 0.006 | 0.75(0.57-1.00) | 0.048 | 0.76(0.57-1.01) | 0.055 | 0.76(0.58-1.01) | 0.06 |
|  | Q5 (6.76654,27.60012) | 0.59(0.44-0.77) | <.001 | 0.65(0.46-0.91) | 0.011 | 0.66(0.47-0.92) | 0.015 | 0.68(0.49-0.95) | 0.024 |
| **CKD stage 4** | |  |  |  |  |  |  |  |  |
|  | Q1 (0.96973,3.01255) | Ref |  | Ref |  | Ref |  | Ref |  |
|  | Q2 (3.03622,4.14082) | 1.50(0.52-4.32) | 0.456 | 0.90(0.27-3.04) | 0.868 | 0.81(0.23-2.89) | 0.744 | 0.77(0.21-2.79) | 0.689 |
|  | Q3 (4.15689,5.23685) | 1.11(0.33-3.67) | 0.866 | 0.65(0.16-2.56) | 0.539 | 0.67(0.17-2.69) | 0.575 | 0.65(0.16-2.62) | 0.548 |
|  | Q4 (5.27378,6.70019) | 1.34(0.41-4.36) | 0.625 | 0.68(0.15-3.04) | 0.614 | 0.65(0.14-3.00) | 0.58 | 0.68(0.15-3.15) | 0.618 |
|  | Q5 (6.81344,12.95331) | 0.74(0.19-2.96) | 0.675 | 0.21(0.03-1.44) | 0.111 | 0.16(0.02-1.19) | 0.074 | 0.17(0.02-1.23) | 0.079 |
| **CKD stage 5** | |  |  |  |  |  |  |  |  |
|  | Q1 (0.62319,2.85547) | Ref |  | Ref |  | Ref |  | Ref |  |
|  | Q2 (3.32170,4.08099) | 0.15(0.03-0.82) | 0.028 | 0.20(0.02-1.86) | 0.158 | 0.10(0.01-1.37) | 0.084 | 0.10(0.01-1.20) | 0.069 |
|  | Q3 (4.23040,5.15346) | 1.08(0.24-4.90) | 0.923 | 1.97(0.34-11.40) | 0.448 | 6.43(0.73-56.68) | 0.094 | 4.45(0.40-49.94) | 0.227 |
|  | Q4 (5.40021,6.62043) | 0.31(0.05-1.89) | 0.203 | 0.94(0.08-11.31) | 0.962 | 1.88(0.15-24.29) | 0.627 | 1.72(0.13-22.61) | 0.68 |
|  | Q5 (6.81746,8.18718) | 0.26(0.05-1.45) | 0.125 | 1.32(0.06-31.17) | 0.863 | 0.51(0.01-24.38) | 0.731 | 0.36(0.01-14.90) | 0.591 |
| **Male** | |  |  |  |  |  |  |  |  |
|  | Q1 (0.49984,3.00996) | Ref |  | Ref |  | Ref |  | Ref |  |
|  | Q2 (3.02091,4.14969) | 0.80(0.56-1.12) | 0.194 | 0.88(0.62-1.25) | 0.475 | 0.86(0.60-1.23) | 0.409 | 0.90(0.63-1.28) | 0.555 |
|  | Q3 (4.15696,5.27337) | 0.57(0.39-0.84) | 0.004 | 0.69(0.46-1.03) | 0.071 | 0.66(0.44-0.98) | 0.042 | 0.74(0.49-1.10) | 0.136 |
|  | Q4 (5.28083,6.76164) | 0.67(0.47-0.96) | 0.029 | 0.81(0.54-1.20) | 0.295 | 0.79(0.53-1.17) | 0.236 | 0.81(0.54-1.20) | 0.291 |
|  | Q5 (6.76681,21.95267) | 0.63(0.44-0.91) | 0.013 | 0.85(0.54-1.32) | 0.469 | 0.86(0.55-1.34) | 0.497 | 0.92(0.59-1.45) | 0.729 |
| **Female** | |  |  |  |  |  |  |  |  |
|  | Q1 (0.51288,3.01571) | Ref |  | Ref |  | Ref |  | Ref |  |
|  | Q2 (3.01620,4.15368) | 0.67(0.49-0.91) | 0.011 | 0.66(0.48-0.91) | 0.01 | 0.67(0.49-0.91) | 0.012 | 0.69(0.51-0.95) | 0.023 |
|  | Q3 (4.15689,5.26939) | 0.80(0.58-1.11) | 0.178 | 0.78(0.56-1.09) | 0.145 | 0.78(0.56-1.09) | 0.149 | 0.78(0.56-1.10) | 0.156 |
|  | Q4 (5.27378,6.76295) | 0.75(0.53-1.05) | 0.093 | 0.72(0.49-1.05) | 0.084 | 0.73(0.50-1.07) | 0.103 | 0.75(0.51-1.09) | 0.127 |
|  | Q5 (6.76654,27.60012) | 0.46(0.30-0.70) | <.001 | 0.42(0.26-0.70) | <.001 | 0.44(0.27-0.72) | 0.001 | 0.46(0.28-0.76) | 0.003 |
| **BMI<25** | |  |  |  |  |  |  |  |  |
|  | Q1 (0.49984,3.01571) | Ref |  | Ref |  | Ref |  | Ref |  |
|  | Q2 (3.01620,4.15368) | 0.65(0.48-0.86) | 0.003 | 0.70(0.52-0.94) | 0.019 | 0.70(0.52-0.95) | 0.023 | 0.71(0.52-0.96) | 0.025 |
|  | Q3 (4.15696,5.27337) | 0.68(0.50-0.93) | 0.015 | 0.75(0.54-1.04) | 0.081 | 0.74(0.53-1.03) | 0.071 | 0.76(0.55-1.06) | 0.109 |
|  | Q4 (5.27378,6.76295) | 0.70(0.51-0.96) | 0.027 | 0.76(0.54-1.08) | 0.131 | 0.77(0.54-1.09) | 0.142 | 0.75(0.53-1.07) | 0.109 |
|  | Q5 (6.76654,27.60012) | 0.50(0.35-0.72) | <.001 | 0.59(0.38-0.91) | 0.016 | 0.62(0.40-0.95) | 0.028 | 0.63(0.41-0.97) | 0.036 |
| **BMI≥25** | |  |  |  |  |  |  |  |  |
|  | Q1 (0.58577,3.01464) | Ref |  | Ref |  | Ref |  | Ref |  |
|  | Q2 (3.01829,4.14969) | 0.88(0.61-1.27) | 0.492 | 0.89(0.61-1.29) | 0.536 | 0.85(0.58-1.24) | 0.399 | 0.99(0.68-1.44) | 0.95 |
|  | Q3 (4.15689,5.26939) | 0.67(0.45-0.99) | 0.045 | 0.68(0.45-1.04) | 0.073 | 0.67(0.44-1.01) | 0.056 | 0.77(0.50-1.16) | 0.21 |
|  | Q4 (5.27742,6.76164) | 0.73(0.49-1.09) | 0.125 | 0.75(0.49-1.14) | 0.179 | 0.75(0.49-1.14) | 0.18 | 0.87(0.57-1.35) | 0.544 |
|  | Q5 (6.77908,21.95267) | 0.64(0.42-0.97) | 0.034 | 0.65(0.40-1.07) | 0.091 | 0.65(0.40-1.08) | 0.094 | 0.81(0.49-1.33) | 0.401 |
